# Multi-OMICs and Genome Editing Perspectives on Liver Cancer Signaling Networks

**DOI:** 10.1155/2016/6186281

**Published:** 2016-06-14

**Authors:** Shengda Lin, Yi A. Yin, Xiaoqian Jiang, Nidhi Sahni, Song Yi

**Affiliations:** ^1^Department of Medicine, Stanford University School of Medicine, Stanford, CA 94305, USA; ^2^Cancer Biology Program, Stanford University School of Medicine, Stanford, CA 94305, USA; ^3^Division of Biomedical Informatics, University of California, San Diego, La Jolla, CA 92093, USA; ^4^Department of Systems Biology, The University of Texas MD Anderson Cancer Center, Houston, TX 77030, USA; ^5^Department of Genetics, Harvard Medical School, Boston, MA 02115, USA

## Abstract

The advent of the human genome sequence and the resulting ~20,000 genes provide a crucial framework for a transition from traditional biology to an integrative “OMICs” arena (Lander et al., 2001; Venter et al., 2001; Kitano, 2002). This brings in a revolution for cancer research, which now enters a big data era. In the past decade, with the facilitation by next-generation sequencing, there have been a huge number of large-scale sequencing efforts, such as The Cancer Genome Atlas (TCGA), the HapMap, and the 1000 genomes project. As a result, a deluge of genomic information becomes available from patients stricken by a variety of cancer types. The list of cancer-associated genes is ever expanding. New discoveries are made on how frequent and highly penetrant mutations, such as those in the telomerase reverse transcriptase (*TERT*) and* TP53*, function in cancer initiation, progression, and metastasis. Most genes with relatively frequent but weakly penetrant cancer mutations still remain to be characterized. In addition, genes that harbor rare but highly penetrant cancer-associated mutations continue to emerge. Here, we review recent advances related to cancer genomics, proteomics, and systems biology and suggest new perspectives in targeted therapy and precision medicine.

## 1. Genetic Alterations in the Cancer Genome: Liver Cancer as an Example

Significant effort has been made to reveal the mutational landscape of cancers. Herein, we use liver cancer as an example to demonstrate recent advances. Primary liver cancer is the sixth most frequent cancer worldwide and a leading cause of death in Asia [1, 2], with hepatocellular carcinoma (HCC) as the most common form, followed by intrahepatic cholangiocarcinoma (IHCC) [2]. Most liver cancers are developed from liver cirrhosis with hepatitis B virus (HBV) and hepatitis C virus (HCV) infections, with alcohol consumption, metabolic diseases, and chemical exposure as major predisposing factors [3–5].

The capacity of next-generation sequencing (NGS) has dramatically increased over the years due to technological advances. Cost per raw megabase of DNA sequence has gone down from over $5000 in 2001 to $0.015 in mid-2015, at a rate faster than Moore's Law [6], allowing more samples to be sequenced in parallel and more powerful statistical analyses to be performed. Whole-genome sequencing (WGS) and exome sequencing have identified various genetic alterations in liver cancer. The first whole-genome sequencing of a HCC genome with HCV revealed more than 11,000 somatic substitutions in the tumor genome and 22 validated chromosomal rearrangements [7]. A later study sequenced 27 HCCs, 25 of which were with HBV or HCV, including 2 sets of multicentric tumors [8]. Multiple chromatin regulator genes, including* ARID1A*,* ARID1B*,* ARID2*,* MLL,* and* MLL3*, were detected in ~50% of the tumors. In addition, HBV integration in the* TERT* locus was frequently observed in a high clonal proportion. Another WGS study of 88 HCC tumors, among which 81 were associated with HBV, found* CTNNB1* to be the most frequently mutated oncogene (15.9%) and* TP53* to be the most frequently mutated tumor suppressor (35.2%) [9]. Integrated analysis of somatic mutations and focal copy-number changes of 125 HCC tumors and whole exome sequencing on 24 of these samples identified 135 homozygous deletions and 994 somatic mutations [10]. New recurrent alterations in* ARID1A*,* RPS6KA3*,* NFE2L2,* and* IRF2* were found in this study. To date, ~1000 HCCs have been sequenced, which provides a mutational landscape of HCC. Most common mutations, including* TERT* promoter mutations (56%),* TP53 *(27%),* CTNNB1 *(26%),* ARID2 *(7%),* ARID1A *(6%), and* Axin1* (5%), as well as key signaling pathways, such as the canonical WNT signaling pathway and the JAK/STAT pathway, were shown to be altered in liver cancers [9, 11, 12].

Moreover, deep-sequencing technologies have greatly facilitated pathogenic analysis of liver cancers stratified by etiology. A recent exome sequencing study on 243 liver tumors identified 161 putative driver genes associated with 11 recurrently altered pathways [13]. Association of mutations and risk factors defined 3 groups of genes centered on* CTNNB1* (alcohol),* TP53* (hepatitis B virus, HBV), and* AXIN1*.* TERT* promoter mutations and* TP53* alterations were associated with early and advanced stages in the tumors, respectively [13].* TERT *reactivation is also associated with HBV infection. Although genome integration is not required for HBV replication, fragments of HBV DNA are found in chronic HBV infections and 85–90% of HBV-related HCC [14, 15]. Most of the integration events result in unidirectional upregulation of genes at the integration sites [16]. Consistent with previous analysis of HBV integration sites using PCR  [17, 18], high-depth genome sequencing of HBV-positive HCC samples identified frequent HBV integration including the* TERT* locus [8, 19].

As the most frequently mutated target, telomerase plays a central role in liver cancers. Telomerase extends the terminal segment of eukaryotic chromosomes known as the telomeres [1, 2, 20]. Normal cells could only undergo a finite number of divisions in culture before entering a senescence state, a phenomenon discovered by Hayflick and Moorhead in the 1960's [21]. In contrast, cancer cells counter the “end-replication problem” by acquiring the capacity to maintain the telomeres. 80–90% of human cancers sustain their telomeres by reactivating telomerase [22]. The catalytic core of the telomerase consists of the catalytic protein component encoded by* TERT* and the RNA component* TERC *[2, 23]. Additional components, such as dyskerin (DKC) and telomerase Cajal body protein 1 (TCAB1), are required for the holoenzyme to function* in vivo *[3, 4, 24–31]. Telomerase expression is primarily controlled by the transcription of* TERT *[32–34]. Most somatic cells do not express* TERT* and lack telomerase activities [35–37]. The connection between telomere regulation and liver cancers was first studied in Japanese patients in the 1990's. Shortening of telomeres was reported in cirrhosis patients over 45 years with viral hepatitis, and telomerase reactivation was also observed in HCC patients [21, 38–41].

An important insight into the mechanism of* TERT* reactivation was discovered in 2013, when two independent studies identified recurrent somatic mutations in the core promoter of* TERT* genes in different melanoma samples [42, 43]. The most prevalent somatic mutations were two mutually exclusive “C>T” transitions at -124 and -146 from the translational start ATG of the* TERT* gene, respectively. These mutations were subsequently identified in a wide range of other human cancers including HCC, glioma, thyroid, and bladder cancers [44, 45]. Additional less frequent mutations were also detected in the* TERT* promoter, including the tandem mutations “CC>TT” at −124~125 and −138~139 bp from ATG, as well as a A>C transversion at −57 bp from the ATG [43]. These mutations created* de novo* binding motif for Ets/TCF transcription factors. A study of 23 human urothelial cancer cell lines demonstrated that these promoter mutations are correlated with higher levels of* TERT* mRNA, protein, telomerase activity, and telomere length [46]. A member of the Ets family, GABP, was found to be recruited to the mutation site to activate* TERT *[47]. Together, recent findings firmly established that the genetic alterations at the* TERT* promoter play a central role for the cancer-specific telomerase activation. In HCC, the -124C>T accounted for 93% of the total mutations detected, and the frequency of -146C>T was 6%. Promoter mutations were identified in 5 out of 20 macronodules of cirrhotic but not in the 69 cirrhotic tissues, suggesting that the* TERT* promoter mutation is an early genomic alteration that transitions liver cirrhosis to carcinogenesis [48]. Interestingly,* TERT* promoter mutations were not detected in the benign hepatocellular adenoma; in contrast, 7/16 (48%) malignant tumors transformed from HCA and 58/106 (55%) of HCCs in normal liver exhibited the mutations, all of which are significantly associated with mutations activating canonical WNT signaling pathway. Thus, telomerase was activated at a later stage of HCC without cirrhosis.

## 2. Multi-Omics to Unravel Cancer Mystery: Evolution of Functional Genomics and Proteomics

**Figure 1:**
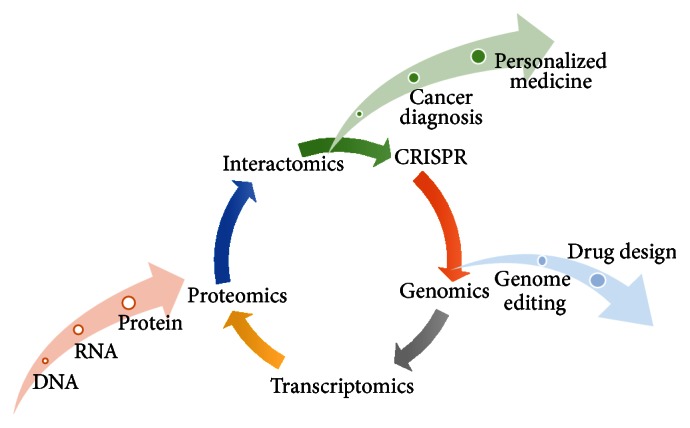
Multi-OMIC systems approach to elucidate cancer signaling networks and precision medicine.

The advent of human genome sequences has changed our ways to address fundamental questions in human cancer. With information available for thousands of genes, the conventional method of studying one gene (or one protein) at a time could now be complemented by more systematic platforms that study multiple or even all genes at large scale. A potential barrier to this prospect, however, is that most genes have not yet been empirically characterized. For most gene products (or proteins) in the proteome there is a lack of functional information that can be obtained or derived from any biological model. Toward this end, in the recent past, high-throughput functional genomic and proteomic strategies have been invented to facilitate the annotation of large numbers of genes. Such “systems biology” approaches aim to generate quantitative and dynamic models and to interrogate key biological processes with holistic insights (Figure 1). Herein we summarize a few such high-throughput genome-wide functional platforms that have been developed [49–53].

### 2.1. Gene Expression

Expression profiling techniques such as microarray and RNA-seq provide an estimate of mRNAs (transcription levels) present under a given condition in a cell- or tissue-specific fashion. Making transcript level measurements under many different conditions defines a “transcriptome” for a given organism [54]. Microarray is based on the hybridization of a cDNA library to a DNA chip to determine relative abundance of usually fluorophore-labeled targets [55]. RNA-seq takes advantage of next-generation sequencing to quantify the amount of RNAs after reverse transcription [56]. With gene expression data, clustering analyses can be performed to group genes that are similarly expressed. These expression profile clusters often contain functionally related genes that are coregulated and could suggest new functional hypotheses for uncharacterized genes in the same clusters. For correlation measurement, Pearson correlation coefficients are often used with proper titration adjustments [57].

Gene expression is thought to be primarily regulated by transcription factor binding at a given time [56]. Recent studies also revealed important roles of lncRNAs [58] and miRNAs [59] in gene regulation. With the facilitation of modern technologies and next-generation sequencing, RNA-seq gene expression can now be easily performed at the single cell level [60]. However, gene expression at the transcriptional levels may not correlate well with the translational levels [61], so protein-centric studies need to take additional proteomic assays for validation.

### 2.2. Proteomics

Numerous proteomics approaches have been developed and applied to study large-scale protein functions. Protein localization mapping projects assign functionally related proteins to the same subcellular compartments at similar times, given their possible involvement in similar biological processes [62]. Reverse phase protein array (RPPA) is a proteomics technology that allows for quantitative protein expression measurement at large scale based on high-quality antibodies [63].

On the other hand, large-scale macromolecular interaction screening tools, such as yeast two-hybrid (Y2H) systems or mass spectrometry (TAP/MS), have been widely used to map protein-protein interaction networks in different species, including human. Physically interacting protein partners are believed to share signaling pathways, GO terms, or memberships in protein families [64, 65]. Functionally related gene products often act as macromolecular complexes and form topological modules in the interaction networks, by which hypothesis of function for many unknown proteins could be formulated.

### 2.3. Data Repositories for Cancer

The Human Gene Mutation Database (HGMD) is a comprehensive repository of germ-line mutations in genes that are causal for, or are associated with, human disease, including cancer [66]. The ClinVar database [67] from NCBI also contains cancer mutation annotations. Mode of inheritance information for each cancer type can be obtained from two databases: Online Mendelian Inheritance in Man (OMIM) [68] and Universal Protein Resource (UniProt) [69].

The Cancer Genome Atlas (TCGA) is a large repository for genetic mutations in more than 30 cancer types, including ~500 patient samples [70]. TCGA is also an enormous resource for profiling of gene expression, copy-number variation, DNA methylation, and so forth [71, 72]. The International Cancer Genome Consortium (ICGC) is a collaborative organization that aims to coordinate large-scale genomic, transcriptomic, and epigenomic data for over 50 cancer types around the world [73]. The Cancer Cell Line Encyclopedia (CCLE) is another collaborative project with a goal of providing comprehensive genomic data and computational analysis for ~1,000 human cancer cell lines [74]. To facilitate the easy use of multidimensional cancer genomic data, cBioPortal was established to provide a web resource for exploring, visualizing, and analyzing molecular profiling data in cancer tissues and cell lines [75]. Furthermore, large-scale phenotypic analysis can also help characterize genes and suggest potential functional descriptions for many unknown genes. By identifying possible phenotypes attributable to disruptions or alterations in specific genes using technologies such as knock-outs or RNAi, genes with similar phenotypes can be found that might function together in common functional pathways in a given cellular context [57, 76].

## 3. An Evolving Systems Biology Toolkit for Better Cancer Precision Medicine

A key leap forward in the development of a cutting-edge cancer research toolkit is to design strategies to flexibly express any genes in the human genome (Figure 1), in order to study them in various cells, under different conditions and in many biological processes of interest. In other words, there is a dire need to develop diverse large-scale functional genomic and proteomic platforms. High-throughput studies [64, 65, 77–82] often require large numbers of protein-encoding genes to be expressed precisely, that is, in-frame without any 5′UTRs, 3′UTRs, or introns, into various expression systems.

### 3.1. High-Throughput Gateway Technology for Functional Studies

Gateway is a modern molecular technology amenable for high-throughput and automated biomedical experiments. Gateway technology is designed for easy transfer of DNA fragments based on site-specific recombination principle [77, 83, 84]. In this big data era, Gateway has emerged as a cutting-edge tool to facilitate large-scale genomic and functional studies, such as mutagenesis, sequence tagging, protein purification, promoter, and RNA analysis. It has been increasingly appreciated and widely adopted in a variety of cancer research areas.

Gateway technology enables convenient DNA transfer, taking advantage of the recombination machinery between the genomes of bacteria and phage. This process is reversible and involves two enzyme mixes (“BP” and “LR” clonase) and a set of recombination sequences (“*att*” sites). The recombination events are described briefly below. (i) Catalyzed by the BP clonase mix, the* attP* site of the phage DNA recombines with the* attB* site from the bacterial DNA, deriving two new sites,* attL* and* attR*. (ii) Catalyzed by the LR clonase mix, the* attL* and* attR* sites recombine in the excision reaction, reverting back to the* attP* and* attB* sites.

When implementing the Gateway technology in molecular biology, a typical “Gateway Cassette” is designed as a module to insert into a vector. The four recombinational sites (*attB*,* attP*,* attL,* and* attR*) are duplicated and modified. In the BP reaction, we start with a “Donor” vector, containing a Gateway Cassette with* P1* and* P2* sites and usually a chloramphenicol resistance selection marker. The* P1* and* P2* sites on the Donor plasmid recombine with* B1* and* B2* sites, respectively, which flank a DNA sequence of interest. In this way, the DNA of interest can be cloned unidirectionally into the Donor vector. The resulting product is known as an “Entry” clone, containing two* attL* sites,* L1* and* L2*.

Gateway Entry clones can be readily transferred via an “LR” reaction into various expression vectors, known as Destination vectors, for downstream functional studies. In the LR reaction, the* R1* and* R2* sites on Destination plasmids recombine with the* L1* and* L2* sites, respectively, on the Entry clones. Many popular prokaryotic and eukaryotic expression Destination vectors are available, such as yeast two-hybrid AD and DB vectors, fluorescence-based PCA vectors, and LUMIER Myc- and flag-tagged vectors for coimmunoprecipitation. In addition, other existing functional expression vectors can be readily converted to Gateway-compatible Destination Vectors, by inserting a Gateway Cassette. With the fast growing of genomic information and larger-scale research nowadays, the Gateway cloning system apparently emerges as a powerful, high-throughput platform compatible with the current research needs. A collection of genes, as Gateway Entry clones, can be transferred at large scale to one or more Destination Vectors in a simple reaction, manually or robotically.

### 3.2. The Human ORFeome: A Versatile Tool for Cancer Research

Large libraries of Gateway Entry clones, encompassing all possible open reading frames (ORFs) [84] in the genomes of many species including humans, are necessary for high-throughput functional studies. Ideally, the human “ORFeome” corresponds to all full-length protein encoding genes, including possible variants and isoforms in different tissues, developmental stages, and across the human population. However, identifying such a comprehensive ORFeome collection is apparently challenging, due to limitations in existing experimental strategies [85].

Initial efforts in the construction of a human ORFeome library took advantage of public collections of human cDNAs, such as the Mammalian Gene Collection (MGC) [86]. Using MGC as template for PCR amplification, ~8000 ORFs were Gateway cloned without containing a stop codon; thus, N-terminal and C-terminal protein fusions can both be feasible downstream in Destination vectors. Because there may be multiple splice isoforms and polymorphic variant ORFs for the same gene, the 8000 ORFs represented ~7000 distinct genes. Clones shorter than 100 nucleotides and clones without complete coding sequences (CDS) available in NCBI were eliminated. Successfully cloned ORFs were consolidated as the first version of the human ORFeome collection (hORFeome v1.1) [87]. In 2007, the human ORFeome v3.1, adding ~4,000 new ORFs, brings the total to 12,212 distinct ORFs, representing 10,214 distinct genes [88]. In 2011, the human ORFeome v8.1 was released, containing 16,172 ORFs mapping to 13,833 distinct genes [89]. This extensive ORF library represents an important resource of single-colony, fully sequence-verified human ORFeome Entry clone collection. This set of ORFs ranges in size from 75 to more than 10,000 base pairs. In addition, an Expression Library version of this hORFeome v8.1 was constructed in a lentiviral expression vector that produces consistent titers and gene expression levels and allows delivery to most cell types [89].

## 4. The Human Interactome: A Scaffold for Functional Proteomics and Evolution in Cancer

Identification of human cancer genes in which mutations are associated with specific clinical manifestations has facilitated our understanding of disease mechanisms. However, like their normal counterparts, protein products of cancer genes do not function in isolation but are part of highly interconnected cellular signal transduction networks (Figure 1) [90, 91].

### 4.1. Literature-Derived Interactome (LDI)

Interactome networks could be derived from literature through two different approaches: text mining and manual curation. Text mining is performed computationally by searching for key words in literature databases, such as PUBMED. Manual curation of literature knowledge involves enormous amount of labor and time. However, certain datasets of human molecular interactions have been curated from the literature and stored in public databases, such as BioGRID [92], CORUM [93], BIND [94], DIP [95], STRING [96], HPRD [97], MINT [98], GeneMania [99], and MIPS [100].

### 4.2. Empirically Derived Interactome (EDI)

Modern molecular biology has brought in many advanced tools for functional studies, but most of them experience limitations when it comes to scale-up to a genome-wide investigation. However, a number of experimental strategies have been employed in large-scale human interactome mapping, such as yeast two-hybrid [79, 101, 102], cofractionation [103], and affinity purification followed by mass spectrometry (AP-MS) [104]. Early efforts using high-throughput systematic yeast two-hybrid platforms have generated preliminary human protein-protein interactome network maps [79, 101]. In 2005, two studies simultaneously reported the first version of human interactome map. 2,754 high-confidence protein-protein interactions among 1,549 proteins were reported in the CCSB-HI1 dataset [79], while 3,186 interactions involving 1,705 proteins were reported in the Stelzl network [101]. A second generation of interactome map was recently published, containing 13,944 interactions among 4,303 distinct proteins [102]. This map covers a vast previously uncharted territory and is 30% larger than the literature of all small-scale studies combined in the past few decades. It is demonstrated to be helpful in predicting novel cancer genes and other disease-associated mechanisms.

Proteome-scale studies of human interactome networks have also been performed using other high-throughput approaches. Based on biochemical fractionation and quantitative mass spectrometry, Havugimana et al. identified a map of 622 protein complexes in human cells. This interactome map profiles 13,993 physical interactions between 3,006 proteins, revealing many interesting biological associations [103]. Lately, another interactome network map systematically charted by affinity purification followed by mass spectrometry (AP-MS) provided another functional view of protein complexes, covering 23,744 interactions among 7,668 proteins with many unexpected hypotheses for previously poorly characterized proteins [104]. Furthermore, another group performed a quantitative network survey to capture human interactome networks with higher resolution in interaction strength and protein abundance [105]. They used quantitative bacterial artificial chromosome with GFP fusion interactomics (QUBIC) and identified 28,504 unique interactions involving 5,462 proteins. It was demonstrated that weak interactions dominate the network and have topological properties.

To assess the specificity of interactome networks, a random subset of interactions is selected typically for an independent and orthogonal validation to confirm the overall quality of the human interaction networks. It is noteworthy that not all the interactions reported in literature are of high quality or necessarily interpreted as “gold standard.” The ones identified by multiple publications or methods tend to be genuine interactions. As expected in any biological assay, the resulting networks exhibit a large fraction of false negatives. To assess the sensitivity of interactome networks, high-confidence subsets of literature-derived interactions can be employed as a comparison for sensitivity measurements.

## 5. Systems Biology Reveals Functional and Evolutionary Insights into Human Diseases Like Cancer

### 5.1. Computational Modeling

Computational modeling has been useful in predicting the functional impact of genes and mutations that are difficult to test experimentally. Polymorphism Phenotyping v2 (PolyPhen-2) [106] was developed to predict the functional significance of a genetic variant based on conservation, protein structure, and other features using naïve Bayes classifier trained by supervised machine-learning. Mapping of genetic variants to Pfam domains (Pfam-A family only) can be performed using the program Hmmer version 3 [107]. The IUPred program [108] can be used to assess the likelihood of residues affected by a genetic variant located in an intrinsically disordered region of the protein. The regular expressions of known eukaryotic linear motifs (ELMs) can be obtained from the ELM database (http://elm.eu.org/). DSSP program [109] can be used to compute solvent accessible area for each residue mutated by a genetic variant. FoldX force-field algorithm [110, 111] can be used to calculate the change in free energy of unfolding (ΔΔ*G*) for all mutations that could be mapped to a published crystal structure from Protein Data Bank (PDB) [112]. For interaction interface analysis, the mutated residues can be mapped onto the available structures by using Mechismo (http://mechismo.russelllab.org/), ProtInDB (PROTein-protein INterface residues Data Base), and PDBePISA (Proteins, Interfaces, Surfaces, and Assemblies) [113] servers. The database of three-dimensional interacting domains (3did) documents and predicts high-resolution structures for domain-domain interactions [114].

Recently, a structure-based prediction of a proteome-wide human protein-protein interaction network was released [115]. Through experimental validation of a subset of interactions, this computationally predicted interactome (CPI) network was considered to be of high quality. HINT (High-quality INTeractomes) is a database that extracts high-quality protein-protein interactions [116]. Clusters of cancer mutations in the human proteome can be identified by mutation3D algorithm [117]. Looking into the future, the union of all LDI, EDI, and CPI interactions reveals more and more comprehensive human interactome networks, and the interaction pairs argue for their potential biological and functional relevance. However, future efforts are still required to interpret condition-specific interactions and to characterize the effects of genomic variation on interaction networks [118], which will in turn generate insights into genotype-phenotype relationships in human.

### 5.2. Computational Modeling and Network Analysis

Given the highly connected nature of molecular signaling network organization in the cell [119, 120], a conceptual framework was developed to illustrate a global picture (known as “diseasome”) of all the known genes involved in human disease. To construct such a “diseasome” network, a compendium of 1,777 human disease genes and 1,286 associated diseases [121] was obtained from the Online Mendelian Inheritance in Man (OMIM) database. In the diseasome network, the human “disease genome” (a long list of known disease genes) was linked to the “disease phenome” (a list of known genetic disorders), deriving a comprehensive set of almost all known gene-disease network associations. This network-based “genome-phenome” profile [122] is a bipartite graph, in which a gene and a disease are linked together if mutations in that gene have been implicated in that disease.

A Human Disease Network (HDN) was derived from the original bipartite “diseasome” landscape [123]. In the HDN network, nodes represent diseases, and edges represent the association between diseases when they share at least one gene in which mutations are associated with both diseases. Overall, 867 of 1,284 diseases have at least one link to other diseases, and 516 diseases form a single connected cluster, the giant component, suggesting that most human diseases share, to some extent, genetic origins. The HDN network is clearly clustered by major disease classes, reflecting visible differences between classes of disorders but commonality in genetic origin within each disease class. Among the most connected diseases is cancer, which is in part due to the many common regulators (such as p53, PTEN, KRAS, ERBB2, and NF1) associated with distinct subtypes of cancer.

Another type of biologically relevant networks concerns disease gene network (DGN) [123]. In the DGN, nodes represent disease genes, and edges represent their association with the same disease. In this network, 1,377 of 1,777 disease genes are connected to at least one other disease gene, and 903 genes form a giant component. The DGN provides a complementary, gene-centered view of the diseasome than the HDN.

Given that interactome networks cover a myriad of genes implicated in human diseases, including cancers, they provide useful insights into possible disease signaling mechanisms. Although existing empirically derived interaction (EDI) networks are far from being complete, the overlap with literature has been shown to be significant [102], demonstrating the high quality of the EDI networks. On the other hand, these networks offer novel biological hypotheses and guide further studies of disease signal transduction in relevant functional contexts. Functional consequences of molecular interactions can be followed up to understand the logic of complex biological networks. Therefore, emerging human interactome networks will eventually facilitate our understanding of human health and disease.

## 6. Novel Therapeutic Strategies and Precision Medicine

A major problem in cancer treatment is to achieve specific killing of cancer cells while preserving normal cells. Cancer genomes vary from individual to individual.

### 6.1. New Promises of Gene Therapy from CRISPR

The idea of gene therapy was proposed in the 1970's [124]. The 90's witnessed the first successful gene therapy treating patients with severe combined immune deficiency (SCID) by modifying cells with retroviruses carrying a functional copy of the mutated gene [125–128]. However, complications mostly due to integration of viral vector to oncogenes led to suspension of many clinical trials [129]. Nevertheless, quite a few gene therapy strategies made steady strides entering the new century, including Gendicine (first gene therapy product approved for clinical use in humans) [130], oncolytic virus talimogene laherparepvec, and the immunostimulant sipuleucel-T.

The breakthroughs in CRISPR (clustered regularly interspaced short palindromic repeats) mediated genome editing technology provide us with unparalleled opportunity to bring precision medicine to the genome level [131–134]. Compared to targeting malfunctioned molecules at the protein level, it allows for restoration of proper spatiotemporal regulation of the functional molecules without concerns for dosage responses and side effects [131–134]. By correcting disease-causing mutations in embryonic stem cells, disease prevention is made possible even before the onset of symptoms [135–138]. As proof of principle studies, CRISPR-mediated mutation corrections have been successfully performed using multiple mouse disease models including hereditary tyrosinemia and muscular dystrophy [135, 136], resulting in reversion and prevention of diseases, respectively. Given its great potential, CRISPR/Cas9 can revolutionize personalized cancer treatment: to model functional consequences of recurrent mutations identified through high-throughput sequencing efforts, to discover cancer drug targets by screening protein domains [137], and to inhibit cancer by inactivating driver mutations [138].

CRISPR/Cas9 system edits the genome by first creating DNA double-strand breaks (DSBs) [139, 140]. When DSBs occur, the cells activate one of the three mechanisms to repair double-strand breaks: nonhomologous end joining (NHEJ), microhomology-mediated end joining (MMEJ), and homology-directed repair (HDR) [139, 140]. End-joining mechanisms are error-prone and often lead to loss of gene function as a result of random insertions or deletions. In contrast, a DNA sequence, which shares homology with the DSB locus, can be used as a donor template for the HDR pathway to precisely modify the DNA sequence [139, 140]. Sequence-specific endonucleases, such as the zinc-finger nuclease (ZFN) and the transcription-activator-like effector nuclease (TALEN), can introduce double-strand breaks at specific sites of the genome, which dramatically favors the process of HDR instead of NHEJ [139, 140]. ZFN and TALEN have greatly facilitated genome engineering in a variety of model systems [22, 141, 142]. However, the difficulties of designing and building endonucleases tailored to specific genes of interests and the relatively low cutting efficiency have limited their applications. In recent years, Type II CRISPR system emerged as a useful tool for genome editing, with major advantages in cutting efficiency and versatility [131–134]. The most commonly used CRISPR system was modified from the CRISPR-associated endonuclease 9 (Cas9) in* Streptococcus pyogenes* (SpCas9) [139, 140]. The recombinant Cas9 system consists of three components: the Cas9 protein, the CRISPR RNA (crRNA), and the transactivating crRNA (tracrRNA). The crRNA and tracrRNA are often cloned into a single chimeric guide RNA, known as single guide RNA (sgRNA), resulting in an easy-to-use two-component system [139, 140]. The specificity of the endonuclease was determined by the complementation of the sgRNA and its 20-nucleotide target sequence in the genome [143]. The genomic target sequence must be immediately upstream of a 5′-NGG protospacer adjacent motif (PAM) [143]. 5′-NAG can also be tolerated as an alternative PAM [144], albeit with reduced cleavage efficiency [145]. Potential limitations of the CRISPR mainly concern the off-target effects. The seed sequence close to the PAM domain carries more weight in target specificity, while the mismatches further away from the PAM domain and towards the 5′-end of the targeted genome sequence could be tolerated to certain degree [145–147]. Efforts have been made to evaluate and improve the fidelity of the Cas9 system. For example, the Cas9(D10A) mutant, which functions as an ssDNAase, can be used with a pair of sgRNAs complementary to opposite strands of the target DNA, in order to reduce off-targets [148–150]. This is because the DSB generated at the desired site require both nicking events, while sites with a single nicking event are primarily repaired by the more precise excision-repair mechanisms rather than error-prone end-joining mechanisms. Similarly, catalytically inactive cas9 (dCas9) can be fused to the cleavage domain of the FokI restriction endonuclease [151, 152]. The simultaneous binding of two fusion proteins (fCas9) to target sites that are 13~18 bp apart is required for the DSBs to occur [151, 152], as the FokI only cleaves DNA when dimerized.

The efficiency of CRISPR-mediated genome editing is context dependent, with low efficiencies being observed at high “GC” regions or those in close proximity to heterochromatin [139, 140]. In such cases, multistep targeting might be required. For example,* TERT* promoter mutations reside in a genomic region with ~80% GC content. A two-step approach was employed to introduce* TERT* promoter mutations into hESCs. First, two Cas9/sgRNAs were used to delete a 1.5 kb region at* TERT* promoter encompassing the mutation spot. Second, a sgRNA against the newly synthesized NHEJ-derived junction was coelectroporated with Donor plasmids containing the deleted region with cancer-associated* TERT* promoter mutations [153]. In another study, a two-step “pop-in/pop-out” strategy was used to create N-terminal tagged* TERT* fusion protein. First, homologous recombination was achieved by CRISPR/Cas9 targeting the translational start site of* TERT*, with a donor template containing both the tag and an eGFP expressing cassette flanked by LoxP sites. Successfully targeted cells were selected by flow cytometry. Second, eGFP cassette was removed by Cre-mediated recombination [154].

A potential limit of CRISPR-gene therapy concerns the delivery methods. Recombinant AAV (rAAV) is widely considered to be an ideal viral vehicle for gene therapy, because DNA cargo can persist as episomes in both dividing and quiescent cells state with minimal genome integration. Even though exogenous DNA carried by rAAV has been shown to be effective in correcting mutations like the* Fah* mutation in the liver [155], CRISPR technology could result in higher efficiency of gene correction, as proof-of-concept studies demonstrated by hydrodynamic injection of Cas9/sgRNA and a single-stranded DNA to correct the* Fah *mutation in hepatocytes via homology-directed repair [135]. However, the size of the widely used SpCas9 (~4.2 kb) is approaching the cargo limit of rAAV (~4.5 kb), leaving little room for modification. Recently, a smaller Cas9 from* Staphylococcus aureus* (SaCas9) was described [156]. The authors packaged SaCas9/sgRNA into a single rAAV vector and successfully targeted the Pcsk9 gene in the mouse liver.

A broad community of stakeholders have collaborated closely to forge ahead with precision therapy, especially CRISPR-mediated genome editing. Academic researchers continue to provide more accurate insights into human genetics and molecular basis of diseases, as well as develop more powerful bioinformatics tools for analyzing data at the genome scale. Diagnostic companies develop better tests based on newest data to achieve greater precision in interpreting the likelihood of patient response to the therapy. Pharmaceutical companies strive to increase CRISPR targeting efficiency and minimize off-targeting effects, develop reliable quality control process, and build platforms for reducing the cost of CRISPR-mediated gene targeting. Hospitals and other healthcare providers should actively adopt new technologies for individualized prevention, detection, and treatment of diseases. Meanwhile, efforts should be made to provide easy health data access and share mechanisms and protect patient privacy and data security, as well as create platforms to engage different stakeholders in precision medicine as collaborating partners.

### 6.2. Cell Transplantation

The advances of genome-engineering techniques, as well as deeper understanding of the expression profiles of stem/progenitor cells, provide better prospects of cell therapy. As mentioned earlier, initial clinical successes by transplanting genetically modified cells to treat SCID provided valuable proof-of-concept. Here, we used hepatocyte transplantation as an example to discuss some of the promises and challenges of cell transplantation.

To date, only a few treatments can increase the life expectancy of liver cancer patients including resection, orthotopic or living donor liver transplantation, radiofrequency ablation/percutaneous ethanol injection, transcatheter arterial chemoembolization, and sorafenib [157]. Although liver transplantation remains the primary therapeutic strategy for end stage liver diseases and acute liver failures, donor shortage remains a primary hurdle. In an aging population, the supply of liver allografts is unlikely to meet the ever-increasing demand. Therapeutic cell transplantations have been brought into preclinical and clinical applications. There are potential advantages of hepatocyte transplantation over liver transplantation, because hepatocyte transplantation is generally considered to be less invasive, and the native livers are not surgically removed to allow other strategies like gene therapy to be performed.

The potential of hepatocytes as the source for cellular therapy has been demonstrated by years of animal experiments. Rodent hepatocytes have remarkable proliferative capacity* in vivo* [158, 159]. Hepatocyte transplantation was effective in correcting metabolic diseases in several rodent models, including the Gunn rat of Crigler-Najjar syndrome type I [160], the* Fah* ^−/−^ (fumarylacetoacetate hydrolase) mouse of tyrosinemia type I [161], the mutant human *α*1-antitrypsin transgenic mouse [162], and the Long-Evans cinnamon rat of Wilson's disease [163], as well as chemically or surgically induced acute liver failures [164, 165].

A major obstacle to overcome for translating animal studies to human patients is how to obtain enough hepatocytes in a safe transplantation route. The most common route of hepatocyte transplantation is through the portal system. Donor hepatocytes that can be safely infused through the portal vein are usually less than 5% of the liver mass (~2e8 cells/kg), in order to avoid portal hypertension, translocation of the cells to systemic circulation, and embolization in the lung [166]. As many as 70~80% of transplanted hepatocytes are entrapped in the portal space or sinusoids and are subsequently cleared by Kupffer cells and granulocytes [167, 168]. The integration of hepatocytes in the recipient liver is inefficient and requires disruption of hepatic sinusoidal endothelia [169]. Moreover, the initial engraftment of transplanted hepatocytes is unlikely to completely reverse the enzyme deficiency. The continuous repopulation of the recipient liver requires substantial selection advantage of the transplanted hepatocytes, which are artificially created in animal models by extensive parenchymal loss or the proliferative deficiency of endogenous hepatocytes. As a result, repeated hepatocyte transplantation may be required to increase the number of engrafted cells. The population of liver stem cells is potentially a good source of cell transplantation, due to its expandability* in vitro* and bipotent differentiation into hepatocytes and cholangiocytes [170–173]. However, recent studies suggest that mature hepatocytes are responsible for most of the liver repopulation during homeostasis and injuries* in vivo *[174–178]. Within the differentiated hepatocytes, there were subpopulations demonstrating higher repopulating capacities than generic hepatocytes [179, 180]. To identify the best population for cell therapy, comprehensive investigation of the heterogeneity of repopulating cells in the liver is required.

### 6.3. Systems Biology and Therapeutic Strategies

Until recently, a paradigm of drug discovery has been that for each disease there will be one (or a few) molecular target(s) that can be affected either positively or negatively by a single chemical compound. This philosophy has clearly been successful for many diseases and has led to the development of “blockbuster” drugs such as the various ACE-inhibitors or Gleevec. However, this one-gene-one-drug approach has given rise to only ~500 drug targets [181] which, after all, represent a tiny portion of the predicted proteome, estimated at ~500,000 proteins taking into account all isoforms and posttranslational modifications [182]. Moreover the “one-gene-one-phenotype” approach is overly simplistic, because one gene can have multiple functions whereas one function can be handled by multiple genes. For example, various “regulatory” proteins such as Ras, Myc, and NF-*κ*B each have disparate functions that are dependent upon cellular context [182]. Clearly, reliance on the “one-gene or one-protein leading to one drug” paradigm will continue to produce useful drugs, but this strategy is increasingly more difficult to implement [183].

Systems biology approaches have been recently applied to enable a holistic view of signaling networks in cancer cells and effectively identify molecular changes in cancer patients (Figure 1) [184]. For instance, global transcriptomic data analyses in B-cell lymphoma from The Cancer Genome Atlas (TCGA) revealed that older patients tend to exhibit decreased metabolism and telomere function, while female patients are likely associated with decreased interferon and PD-1 signaling [185]. In addition, a critical leap forward in proteomics is the gene-centric Human Protein Atlas for expression profiles [186], which resolves tissue-specific proteome variation of the human body [187] and provides significant insights into cancer pathology [188–190].

An alternative strategy is to understand the structural features and properties of molecular and physiological networks [182, 191]. Although this approach may not have immediate returns in terms of successful deployment of useful drugs [182], it will, in the long run, lead to better understanding of how to model networks and how to use those models for* in silico* studies [191]. Examples are how genetic polymorphisms affect responses to individual drugs [192] and how network interactions can be manipulated and altered by the actions of oncogenes and tumor suppressor genes on one side or by pharmacological intervention on the other [193]. A major hurdle to be overcome is the identification of cellular networks and all of their constituent units, along with an understanding of the signaling within networks and between/among networks.

## 7. Big Data Management and Security of Medical Information 

The significant improvement of sequencing technologies makes human genomic data increasingly affordable and available in the era of precision medicine [194]. This paper discusses heavily how massive human genomic data open the door to big data science and speed up discoveries. Despite encouraging future, there are also emerging problems with respect to storing, sharing, and analyzing big human genomic data. The recent NIH data sharing policy change allows users to store and analyze human genomic data using cloud-computing services, which address some of the issues. But on the other hand, the privacy challenge becomes more prominent with cloud computing as owners lose the full control of the data. It becomes more complicated as copies of data can be stored in a distributed file system or automatically backed up by the cloud service provider. Without necessary protection, it is risky to use the cloud for handling human genomic data, of which information leakage can lead to reidentification [195–199] and might negatively impact patients. The NIH Security Best Practices for Controlled-Access Data Subject to the NIH Genome Data Sharing (GDS) Policy also states that researchers and their institutions are accountable for ensuring the confidentiality of human genomic data, instead of the cloud service provider. There is an imperative need to develop practical and rigorous privacy protection methods to alleviate the technical burden from human genomic researchers. Several recent surveys [197, 200] discussed the relevant techniques. But it remains unclear how these techniques will perform when applied to real human genomic data. There is a lack of direct comparison of different methods in real-world scenarios. Some recent efforts between the computer science community and the biomedical informatics community to jointly tackle the computation and privacy challenges seem promising [201] and more collaborations are necessary to push the fronts.
